# Exploring perspectives of adolescent mothers on the support needed to mitigate their psychosocial challenges within a district in Lesotho

**DOI:** 10.4102/phcfm.v18i1.5352

**Published:** 2026-06-30

**Authors:** Liketso D. Mokhoele, Nombulelo V. Sepeng, Felicia O. Meno

**Affiliations:** 1 Department of Nursing, Faculty of Health Sciences, University of Pretoria, Pretoria, South Africa

**Keywords:** adolescent mothers, psychological challenges, social challenges, perspectives, Lesotho

## Abstract

**Background:**

Lesotho experiences a high rate of adolescent pregnancies, resulting in a significant number of adolescent mothers. The literature indicates that adolescent mothers need support as they face numerous psychosocial challenges, such as depression and stigmatisation. However, there is limited evidence on the perspectives of adolescent mothers regarding the support required to mitigate their psychosocial challenges in Qacha’s Nek district, Lesotho.

**Aim:**

To explore and describe the adolescent mothers’ perspectives on the support needed to mitigate their psychosocial challenges in Qacha’s Nek district, Lesotho.

**Setting:**

This study was conducted at three selected health facilities in Qacha’s Nek district, Lesotho.

**Methods:**

An exploratory, descriptive, contextual, qualitative design was employed. Fifteen adolescent mothers were selected using purposive sampling. Semi-structured interviews were used to collect data, which was analysed through thematic analysis.

**Results:**

The study identified four themes: psychological support, social support, practical support needed by adolescent mothers, and the benefits of psychosocial support for adolescent mothers. The support needed included strengthening family involvement, financial aid, occupational and counselling support, among others, which are important for reducing depression, meeting daily needs and alleviating other psychosocial challenges of adolescent mothers.

**Conclusion:**

Overall, this study illustrated that adolescent mothers face a lot of psychosocial challenges daily. The findings suggested that they need psychological, social and practical support to address those challenges, which may ultimately improve their well-being together with their children.

**Contribution:**

This study offers critical insights, directly from adolescent mothers themselves, into the psychosocial support they require. It identifies the gaps in family support and community-based psychosocial care within the district. It also offers practical guidance to district health managers and other stakeholders to strengthen existing support structures such as the integration of mental health services that respond directly to the expressed needs of adolescent mothers.

## Introduction

Adolescent motherhood is a major global concern that poses unique psychosocial challenges, significantly affecting both young mothers and their children. Each year, an estimated 21 million adolescent girls aged 15–19 years in developing regions experience unintended pregnancies, which result in approximately 12 million births. Sub-Saharan Africa has the highest adolescent birth rate worldwide, at 97.9 births per 1000 women in 2023.^1^ In Lesotho, the adolescent fertility rate is 77 births per 1000 women aged 15–19 years.^2^ The overall fertility rate is higher in rural areas, where this study was conducted. According to the 2023–2024 Lesotho Demographic and Health Survey, approximately 17% of adolescent women (ages 15–19 years) in the country have a history of pregnancy.^2^

This figure is primarily composed of the 14% who have already given birth and the 3% who were pregnant at the time the survey was conducted.^2^ Additionally, 1% of young women in this age group have experienced a pregnancy loss.^2^

Previous research has established an empirical link between a lack of social support and negative psychological health outcomes in adolescent mothers.^3^ According to Anbi et al.,^4^ adolescent mothers may be uniquely vulnerable to such challenges because adolescence itself is a transitional phase, characterised by profound psychological, biological and social changes, and teen pregnancy and parenting disrupt these ongoing processes of growth and development. However, healthcare services often prioritise the medical well-being of the mother and baby, overlooking their complex social and psychological needs. It is at this precise juncture that their mental health is most vulnerable, making holistic support essential. Despite this critical need, persistent gaps in the healthcare system have left adolescent mothers’ psychological health issues largely unaddressed.^4^

Adolescent mothers are at an increased risk of psychological issues such as guilt, stress, despair, low self-esteem, and depression that may lead to suicidal thoughts from feeling overwhelmed by motherhood, particularly when lacking support from partners and families.^5,6^ The lack of support also elevates risk for developing postpartum depression and post-traumatic stress disorder, with risk factors including inadequate income, unwanted pregnancy, lack of spousal support and infant restlessness.^4^

According to Muthelo et al.,^7^ adolescent mothers also encounter significant social challenges, including stigmatisation, discrimination and rejection from partners, friends, families and communities. They are frequently forced to leave school because of financial constraints and struggle to balance parenthood with their academic responsibilities.^7^

To overcome psychosocial challenges, adolescent mothers need suitable, tailored support that directly addresses their specific emotional and social needs. Mabetha et al.^3^ indicate that social support, encompassing emotional, instrumental, affectionate and tangible assistance, such as childcare assistance and provision of basic needs from partners, peers and family, plays a significant role in enhancing their psychological well-being. This support, characterised by meeting individual needs through recurring interactions, substantially influences mothers’ psychosocial health.

Financial support is a critical need for adolescent mothers. A Rwandan study highlighted the short- and long-term financial hardships young mothers face, which affect their ability to provide for themselves and their children.^8^ Academic support is also crucial. According to Babedi,^9^ there is a need to improve the implementation of school support programmes, including a psychosocial support framework to facilitate adolescent mothers’ reintegration into education. A multi-sectoral approach involving family, community organisations, healthcare providers and policymakers is necessary to create supportive environments for adolescent mothers.

Although few studies have been conducted on this subject, most have approached it from the perspectives of various stakeholders rather than from those of adolescent mothers themselves. Against this background, this study aims to explore and describe the perspectives of adolescent mothers in Lesotho’s Qacha’s Nek district on the support needed to mitigate their psychosocial challenges.

## Research methods and design

### Study design

A qualitative, exploratory, descriptive and contextual design was used for this study. Qualitative research investigates the subjective meanings, experiences and understandings of individuals.^10^ And this design was deemed appropriate for this study because it allowed the researcher to explore, describe and understand adolescent mothers’ support needs within their specific context, ultimately generating rich and relevant findings. An exploratory approach was employed to examine adolescent mothers’ perspectives on the support needed to address their psychosocial challenges in Qacha’s Nek district, Lesotho, a topic that has received limited coverage in the existing literature.

A descriptive design was used to describe these perspectives on the required support Polit and Beck.^11^ Descriptive design allowed the researcher to capture a detailed account of the support adolescent mothers truly need. By presenting the participants’ perspectives or experiences in their own words, the findings remain grounded in their actual lives rather than being shaped by pre-existing theories. Contextual design focuses on understanding a phenomenon within its specific environmental, situational, cultural and social setting.^10^ This approach was applied to explore and describe adolescent mothers’ views on the support needed to alleviate their psychosocial difficulties in the context of Qacha’s Nek district, Lesotho.

### Study setting

The study was conducted in Lesotho’s Qacha’s Nek district, an area of 2349 km^2^ with approximately 80 000 residents. This setting was selected because it is a rural area with high adolescent birth rates. This place is an underdeveloped area with limited access to healthcare, resources, education and social services, increasing the vulnerability of adolescent mothers and providing relevant context for the study. Research took place across three rural health centres selected.

One centre is located in the remote area of Melikane, on the district’s outskirts, serving approximately 24 villages with a target population of 5036 in 2023. Another is a government facility in Ha-Mohlapiso village, serving about nine villages and targeting a population of 2717 in 2024. The final centre is a Christian Health Association of Lesotho (CHAL) institution, overseen by the Lesotho Catholic Bishops’ Conference (LCBC).

Situated in the western foothills of Souro in the Tswelikana Council, it is 52 km from the district referral hospital and serves 12 villages with an expected population of 3350 in 2024.

### Study population and sampling strategy

The study population consisted of adolescent mothers. Fifteen participants were purposively selected for this study. The inclusion criteria were adolescent mothers aged 13–19 years who attended clinic services at three selected Health Care Centres in Qacha’s Nek district during the data collection period. These mothers were actively involved in parenting children aged 3 months to 5 years.

Available adolescent mothers who attended Maternal and Child Health (MCH) services, such as immunisations for children under five and general consultations at the selected centres, and who were willing to participate were included. Data collection continued until data saturation was reached, at which point 15 participants had been interviewed.

### Data collection

The researcher collected data using semi-structured, face-to-face individual interviews. Participants were asked: *What are your perspectives regarding the support needed to address your psychosocial challenges in Qacha’s Nek district in Lesotho?* To encourage detailed responses, the researcher used active listening techniques, such as probing, paraphrasing and nonverbal cues, including nodding. Rapport and participant comfort were prioritised, with the researcher attentively observing facial expressions and offering verbal reinforcement to convey genuine engagement.

Data were gathered and preserved through audio recordings, taken with participant consent and field notes. Eligible adolescent mothers attending MCH services, such as immunisations for children under five and general consultations, were recruited by clinic managers, based on their willingness to participate. Interviews were held in private clinic rooms to ensure privacy and confidentiality. Conducted in Sesotho to allow free expression, the interviews were audio-recorded, transcribed verbatim, translated into English and then back-translated into Sesotho to preserve content fidelity. Each interview lasted 30 min to 60 min.

### Data analysis

The researcher applied thematic analysis to examine the qualitative data. As defined by Braun and Clarke,^12^ this method involves identifying and interpreting recurring patterns or themes. This study followed their six-step framework: Data familiarisation, initial coding, searching for themes, reviewing themes, defining themes and final reporting. In this study, data were analysed inductively using ATLAS.ti (Version 25), following the specific steps outlined: (1) A ‘New Project’ was created to include all documents, quotations and codes; (2) the documents (transcripts) were prepared for import and were imported into the programme; (3) to create a textual quotation, the text segments were selected in the document. ‘Coding’ was chosen from the selected context menu, and the quotation appeared in the quotation drop-down list; (4) ‘Open Coding’ was used to create a new code associated with existing quotations or text segments. A new code was added to the code list, and if needed, a new quotation was added to the quotation list; (5) code groups were created. Codes were assigned to code groups, which represented the themes and codes of the subthemes in the table with findings; and (6) the co-coder and researcher discussed and reached consensus on the final themes and subthemes.

### Trustworthiness

The trustworthiness of this qualitative study was established through credibility, dependability, confirmability, transferability and authenticity.^11^ Credibility was achieved through semi-structured interviews, rapport-building with participants and continued data collection until saturation was reached. Member checking was also conducted, allowing participants to verify and clarify their responses, thereby enriching the data.

Dependability was addressed by providing a detailed account of the research methodology, including its objectives, data collection and analysis techniques. An audit trail was maintained through comprehensive documentation of the research process and analytical decisions.

Confirmability was ensured by aligning the research objectives with the interview questions. It was further supported by face-to-face semi-structured interviews, multiple data collection tools, such as audio recordings and field notes, and by playing back recordings to participants.^13^ Transferability was achieved through purposive sampling, which incorporated a diverse range of participant perspectives. A thick description of the research context was provided, and it was clarified that the findings are not generalisable to other settings.^14^

Authenticity was maintained by presenting direct quotations and participant narratives and through collaboration with experienced co-researchers.

### Ethical considerations

Ethical approval for the study was granted by the University of Pretoria, Faculty of Health Sciences (ethical clearance number 726/2024) and the Lesotho Ministry of Health Research Coordinating Unit.

Participation was voluntary. All potential participants aged 18 years and older provided informed consent. For those under 18 years, assent forms were signed by the minors, while their parents or guardians provided consent. Participants were treated equally and without discrimination.

Because of the sensitive nature of the research, a qualified psychologist was available on-site for emotional support. To ensure privacy, interviews were conducted individually in a closed office. All data were handled with strict confidentiality and were accessible only to the research team. Physical data, including field notes and consent forms, were stored in a locked cabinet. Digital data, such as audio recordings, were kept on password-protected computers. Data were permanently deleted using specialised software once it was no longer needed. The study was guided by the principles of autonomy, beneficence, self-determination, justice, confidentiality and the right to privacy.

## Results

This study explored the perspectives of 15 participants from three selected health centres. Using semi-structured interviews, it examined adolescent mothers’ views on the support they need to address their psychosocial challenges. The findings are presented according to demographic information and the identified themes and subthemes from the data analysis. Participant quotations are used to support the results. The demographic data are displayed in Table 1, and the themes and subthemes are shown in Table 2.

**TABLE 1:** Demographic information of the participants.

Participants	Age (years)	Number of children	Health facility
A	19	1	Health Care Centre 1
B	19	1	Health Care Centre 1
C	18	1	Health Care Centre 1
D	18	1	Health Care Centre 1
E	19	1	Health Care Centre 1
F	19	1	Health Care Centre 2
G	17	1	Health Care Centre 2
H	17	2	Health Care Centre 2
I	19	1	Health Care Centre 2
J	19	1	Health Care Centre 3
K	16	1	Health Care Centre 3
L	19	1	Health Care Centre 3
M	15	1	Health Care Centre 3
N	19	1	Health Care Centre 3
P	19	1	Health Care Centre 3

**TABLE 2:** Support is needed by adolescent mothers to mitigate their psychosocial challenges.

Themes	Subthemes
Theme 1: Psychological support required by adolescent mothers	1.1 Counselling
Theme 2: Social support required by adolescent mothers	2.1 Occupational support 2.2 Support regarding custody and paternity issues 2.3 Healthcare support 2.4 Support from others 2.5 Educational support
Theme 3: Practical support required by adolescent mothers	3.1 Support for daily living requirements 3.2 Financial support 3.3 Support for the children of the adolescent mother
Theme 4: Benefits of psychosocial support for adolescent mothers	4.1 Meeting daily living requirements 4.2 Peace of mind and relief of stress 4.3 Independence 4.4 Prospects for a better future

### Theme 1: Psychological support required by adolescent mothers

In this study, adolescent mothers expressed that they need psychological support to manage their psychosocial challenges. This theme, however, yielded only one subtheme: counselling. The subtheme is detailed and is substantiated by direct quotations from the participants.

#### Subtheme 1.1: Counselling

The adolescent mothers expressed that counselling was essential as a form of psychological support to help them cope with their psychosocial challenges. They described a need for someone to talk to and for a space where they could express themselves openly. They believed that such support would play a vital role in managing difficulties like stress. The following statements confirm this:

‘There should be counselling classes where a person can speak freely with others. I do not think any other support can help relieve stress besides that. It is the only solution that allows people to talk openly, as opportunities to do so are rare. The counselling should be held at the clinic. Specific days and hours should be allocated to these sessions.’ (Participant A, Health Care Centre 1, 19 years old)

Another participant indicated:

‘I don’t know, but I think the professional counsellors – I’ve heard that there are counsellors, nutritionists, and others in the Ministry of Health. It should be done in our villages; they usually visit us and organise gatherings for children’s vaccinations, so they should also come with the aim of counselling. They should offer us psychological support until our minds feel refreshed, free of stress or worries. This will reduce thoughts of killing ourselves and the idea of abandoning our children. I believe the main cause of our negative thoughts is that we are often alone with the children. When we go outside, the community discriminates against us. Such counselling would empower us.’ (Participant H, Health Care Centre 2, 17 years old)

### Theme 2: Social support required by adolescent mothers

This study revealed that social support is essential for adolescent mothers to mitigate psychosocial challenges.

This central finding was supported by several emergent subthemes: occupational support, support concerning custody and paternity issues, healthcare support, support from others and educational support.

#### Subtheme 2.1: Occupational support

Occupational support emerged as a pressing need for adolescent mothers. Given the high unemployment rate among both educated and uneducated youth, participants expressed a direct need for jobs. They stated that earning a salary is essential to achieve financial stability and provide care for their children.

The following statements confirm this:

‘Having a job could help me care for my baby and look after myself. Even other teenagers are out there, if they have jobs. I have noticed that other teenage mothers who gave birth on the same day I did need things to just come to them without working for them. In life, you need to have something to do. People want money to flow in, but they don’t understand how it works. So, for me, I need a job to look after my child and to organise everything in my life.’ (Participant E, Health Care Centre 1, 19 years old)

Another participant supported:

‘Adolescent mothers face many challenges. Some have academic certificates but struggle to find jobs. They are unemployed and stay at home. These are the people who require a lot of support, which the government can provide if possible. Most of these needs can be met by the government. The government should provide jobs for mothers like me who are struggling. I would love to be given a job because I currently sit at home all day with nothing to do.’ (Participant G, Health Care Centre 2, 17 years old)

Another participant added:

‘The support available is the social grant I mentioned. If we can’t get help through a social grant, we could be offered jobs. A stable job, not this unpredictable one. I need a job that pays a monthly salary. With the work I am doing now, a month can pass without any clients.’ (Participant H, Health Care Centre 2, 17 years old)

#### Subtheme 2.2: Support regarding custody and paternity issues

Adolescent mothers need support with custody and paternity issues, as they face complex challenges that heighten their psychosocial stress. Participants shared that the fathers of their children often refuse to take responsibility and offer no support. For this reason, they seek assistance with DNA testing to secure child support and ensure the father’s involvement in their child’s life. Others need help navigating the court system to obtain full custody.

This was demonstrated by the following quotations:

‘The support that can be given to adolescent mothers is that if the father, who had previously denied responsibility for the pregnancy, wishes to take the baby after birth, the courts should listen to the mothers, support them, and grant them full custody of the child. The courts should aid mothers and understand why they may not want the father’s family to take their baby. This is because the mother would have faced many challenges. Once you become pregnant, the boy may tell you that he is not responsible for the pregnancy. He might claim he was not the only boyfriend. If you ask him who the other boyfriends were, he might fail to answer. Support can be provided to address such issues.’ (Participant B, Health Centre 1, 19 years old)

Another adolescent mother added:

‘Yes, fathers who refuse to support their children should be punished. The law should be strict in enforcing that fathers take responsibility for their children. No child should grow up without a father’s presence because we feel overwhelmed by raising children alone. I believe that is the main support we need. If necessary, the government should assist with DNA testing. Alternatively, the person requesting DNA evidence should cover the cost.’ (Participant H, Health Centre 2, 17 years old)

Another participant added:

‘Regarding that issue, I believe the mothers should report the fathers’ names so they can be dealt with appropriately. The police should arrest them and instruct them to support their children because when the children are older, the fathers will want to be involved, even though they did not play a role in raising them.’ (Participant I, Health Care Centre 2, 19 years old)

#### Subtheme 2.3: Healthcare support

Adolescent mothers expressed a need for healthcare support to manage their psychosocial challenges. They described difficulty accessing services because of the long distances to facilities and a lack of money for transport.

To overcome these barriers, they want community-based healthcare. This comprehensive care should include family planning and child vaccinations:

‘The village health workers should always carry medication with them. Since many children in the villages fall ill, we should obtain medicines from the village health workers whenever necessary. Sometimes, a child may fall ill either during the day or at night. The mother might not have enough money for transport, especially as we live very far from the clinic. That can affect her psychologically and cause her stress.’ (Participant A, Health Care Centre 1, 19 years old)

Another participant mentioned:

‘Some people live far from the clinic, across the river. The health facility can send two nurses to visit and help them in their communities. They could gather in one village where the nurses will assist them. Assistance with child immunisations and providing medical consultations to sick children. Nurses should continue assisting even during disease outbreaks in those villages.’ (Participant B, Health Care Centre 1, 19 years old)

The other participant added:

‘I believe we should receive support in the form of education on how to avoid having more children while the other is still young. I think we should be provided with contraceptive products until we are in stable marriages.’ (Participant N, Health Care Centre 3, 19 years old)

#### Subtheme 2.4: Support from others

The findings of this study demonstrated that adolescent mothers require support from others, such as parents and community members. They need assistance with childcare to ease the psychosocial challenges of early parenthood. They mentioned the following:

‘The other necessary support is that the father of the child should attend the usual clinic visits with the mother and participate in counselling classes that I have already mentioned with her. This will help the father better understand and assist the baby if something happens while he is babysitting alone. The father should go to the clinic with the mother so he can hear what is discussed there. This will ensure he knows the right things to do to help the baby if it needs assistance.’ (Participant A, Health Care Centre 1, 19 years old)

Another participant supported:

‘The only support I require is the support from my baby daddy. That is the support I need.’ (Participant E, Health Care Centre 1, 19 years old)

Another participant added:

‘We are already receiving support in our communities. In my village, we are encouraged to plant vegetables in the gardens. The support we need from our communities is that they do not ridicule us. We should be treated equally with others. We should not be judged and laughed at by society. We should be accepted and supported, even by our parents; my parents abandoned me. I wish parents could stop abandoning their adolescent girls when they have babies. Our families should give us support because if they don’t, we end up with psychological stress. I can find my life in danger.’ (Participant H, Health Care Centre 2, 17 years old)

#### Subtheme 2.5: Educational support

Adolescent mothers stressed their need for educational support to help them navigate their psychosocial challenges. Most had left school after becoming pregnant but still expressed a strong desire to resume their studies. They believed that access to education would empower them to achieve their goals. They also highlighted the need for flexible schooling options to balance their parental duties with their educational aspirations. Adolescent mothers expressed the following:

‘I would appreciate support to go back to school. I will be repeating Grade 11.’ (Participant J, Health Care Centre 3, 19 years old)

Another participant added:

‘They need to return to school. One will not face many challenges there, as they do when staying at home, where they are often stressed by issues such as food shortages.’ (Participant M, Health Care Centre 3, 15 years old)

Another participant supported:

‘We need to be allowed to learn so that we can achieve our dreams again. There should be a school where we can attend classes on weekends, as mothers, or at any other time, which would be perfect for our tutor.’ (Participant P, Health Care Centre 3, 19 years old)

### Theme 3: Practical support required by adolescent mothers

Practical support emerged as a crucial need for adolescent mothers. This theme was supported by several distinct subthemes: assistance with daily living requirements, financial aid, support for the children of adolescent mothers, the role and involvement of support providers and the frequency of practical support.

#### Subtheme 3.1: Support for daily living requirements

Adolescent mothers expressed that to meet their psychosocial challenges, they require greater support in managing the demands of daily life. They specifically emphasised their need for help securing essential items, such as food and toiletries, for both themselves and their children. The following direct quotations confirm this finding:

‘In terms of food, I can appreciate food donations, which are usually given to others. Things like maize meal and items that can be eaten with soup, because they last longer.’ (Participant D, Health Care Centre 1, 18 years old)

Another participant mentioned this:

‘I believe we can be supported through donations, as adolescent mothers, our children should receive donations. Donations of food, such as maize meal and vegetables. They can buy any food items. We are still young, and we experience menses; we need assistance to maintain hygiene and cleanliness. We require sanitary pads, soap, and other hygiene items.’ (Participant L, Health Care Centre 3, 19 years old)

#### Subtheme 3.2: Financial support

Participants described financial support as a vital form of assistance, noting that it would help address many of their underlying psychosocial issues. They explained that financial difficulties limit their access to education, healthcare and other essential resources, which, in turn, generates significant stress. To ease this burden, adolescent mothers proposed initiatives such as direct government aid and sponsorships for their continued schooling.

The following quotations confirm this finding:

‘I need assistance with school fees. I used to attend school in Maseru. I don’t know how much the school fees are here in Qacha’s Nek. I don’t recall exactly, but I believe it was around M2000.00 annually. The help can come from existing organisations that support other students.’ (Participant D, Health Care Centre 1, 18 years old)

This was alluded to by another participant:

‘If someone could sponsor me to enrol in a design school, I could use the skills to start my own business. We often suffer, and we need money for things like pads. We can receive money for pads, cosmetics, and washing powder.’ (Participant F, Health Care Centre 1, 19 years old)

Another adolescent mother added:

‘I am not sure, but I believe we should receive a social grant similar to the system in South Africa. If the government considered this and provided us with funding, it would benefit us greatly. I have heard that in South Africa, when a child is born, they are given money until they turn 18. I am not entirely certain, but I think children receive money the same way elderly people do here in Lesotho. I am not sure, but I believe we should be given a social grant just like in South Africa. If the government were to consider this and support us, it would help us a lot.’ (Participant H, Health Care Centre 2, 17 years old)

#### Subtheme 3.3: Support for the children of the adolescent mother

Adolescent mothers need support for their children, as this can greatly help alleviate their psychosocial challenges. They expressed a need for nappies and food for their children. They stated the following:

‘I have a problem with Pampers for the baby. They are expensive, and you will find that you do not use one per day. You use many of them, and then they run out very quickly. I need to be supplied with Pampers and other baby essentials.’ (Participant D, Health Care Centre 1, 18 years old)

Another participant mentioned:

‘I would like some food for my child. He eats soup and noodles. He also drinks milk. Additionally, he needs clothes.’ (Participant M, Health Care Centre 3, 15 years old)

### Theme 4: Benefits of psychosocial support for adolescent mothers

The benefits of psychosocial support for adolescent mothers emerged as the final theme. Subthemes included meeting daily living requirements, peace of mind and stress relief, positive psychological effects, independence and prospects for a better future.

#### Subtheme 4.1: Meeting daily living requirements

The study found that one advantage of psychosocial support for adolescent mothers is that it helps them meet their daily living needs. The adolescent mothers stated that the support would help them obtain nutritious food, avoid illness and care for their children. The following statements support this:

‘That money will make a big difference because I will know I will receive government funding each month. I will be able to buy nutritious food for my children. I will also buy food for myself so I can eat healthily as a breastfeeding mother. At the clinic, we are taught to eat healthy, nutritious food. So, that money will help us buy proper food, clothes, and nappies.’ (Participant H, Health Care Centre 2, 17 years old)

Another adolescent mother added:

‘It will also help us to look like others and improve our lives. We will use the employer’s money to buy two or three essentials for survival. It will help us maintain hygiene and cleanliness. It will help us avoid illness because we will be bathing.’ (Participant L, Health Care Centre 3, 19 years old)

#### Subtheme 4.2: Peace of mind and relief of stress

The study’s results showed that another benefit of psychosocial support for adolescent mothers is greater peace of mind and reduced stress. With access to emotional support and counselling, they are better equipped to handle the challenges of parenthood. They feel calmer and generally more at ease.

This is what some of the participants mentioned:

‘Since everyone will be talking and expressing emotions, as a person talks and shares her feelings, she will feel better inside. She will feel liberated. When someone can speak freely, she will feel much less emotionally overwhelmed. Even though her problems will not vanish, she will feel considerably better. She will be able to share with other mothers.’ (Participant A, Health Care Centre 1, 19 years old)

Another participant mentioned:

‘The stress levels will decrease because they will feel relief when someone is talking to them. It helps me in that I don’t have to worry about my baby and abandoning him because he is lacking something. If my baby were lacking, I would always be worried.’ (Participant C, Health Care Centre 1, 18 years old)

Another participant alluded:

‘They will improve. Receiving salaries from jobs will reduce stress and free their minds. Jobs will positively affect their lives because many worry about being unemployed, even though they went to school and earned certificates. It helps me greatly because I know I can rely on them for help since I am not working. I have not found a job anywhere.’ (Participant G, Health Care Centre 2, 17 years old)

#### Subtheme 4.3: Independence

Participants noted that psychosocial support is essential to cultivating their independence, adding that it would empower them to advocate for themselves and reduce their reliance on others.

To confirm this finding, two participants said:

‘It will help them because, in the end, they will be able to stand up to their parents. Even if the parents try to force them into marriage just because they are the father of their children, they will be able to refuse. They will be able to be independent and refuse to marry someone who denies being the father of their children.’ (Participant B, Health Care Centre 1, 19 years old)

This was alluded to by another participant:

‘They will earn their own money and run their errands without asking others for money. Since I will be earning some money, I believe many of my challenges will be resolved, as they all require money.’ (Participant I, Health Care Centre 1, 19 years old)

#### Subtheme 4.4: Prospects for a better future

The results highlighted that psychosocial support would enhance adolescent mothers’ prospects for a better future. Such support would allow them to continue their studies, thereby improving their chances of securing better jobs and building a more prosperous future alongside their children:

‘It will also help us because no child will grow up being told that he or she does not have a father or that a woman cannot have a child alone. Our children will not be raised to believe that their fathers are deceased when they are actually alive. It will benefit not only us but also the children, as both parents will be present in their lives.’ (Participant H, Health Care Centre 2, 17 years old)

Another participant added:

‘Once we start learning, we can advance our studies until we achieve our dreams. We would be able to fulfil our dreams. If I wanted to be a professional teacher, I would study until I am qualified.’ (Participant P, Health Care Centre 3, 19 years old)

## Discussion

This study aimed to explore adolescent mothers’ perspectives on the support needed to alleviate their psychosocial challenges in Qacha’s Nek, Lesotho. Adolescent mothers face significant psychological difficulties in meeting the demands of childcare and require support to overcome these challenges.

The findings indicate that three forms of support are crucial: psychological, social and practical. Psychological support, especially emotional support, is vital as it acts as a buffer against psychological distress.^15^ A significant finding was the expressed need for counselling. Counselling provides essential emotional support by offering a safe and confidential space for adolescent mothers to openly discuss their personal struggles and address emotional issues. This is particularly important given the social discrimination they often encounter, which can hinder their ability to seek external support and interact within their communities. The current shortage of mental health support in existing health facilities highlights a significant gap Mizero et al.^8^ As the findings suggest, mental health services should be integrated into routine maternal health care to effectively support the psychological well-being of adolescent mothers.

Adolescent mothers require various forms of social support to effectively manage their challenges. A key area of need is occupational support, which can help ease their significant financial hardships. They also frequently need assistance with custody and paternity issues, particularly when fathers deny responsibility for their children. This aligns with findings from a Rwandan study that revealed many adolescent mothers face abandonment not only by their own parents but also by the men who impregnated them Mizero et al.^8^

The demanding nature of child-rearing underscores the importance of both parents’ involvement. In some African cultures, the payment of ‘damages’ is viewed as an acknowledgement of paternity, yet most participants did not experience paternity.^15^ They noted that this process offers their families a sense of dignity and some reassurance of the father’s commitment to supporting the child. Consequently, adolescent mothers expressed a need for legal support to obtain sole custody, assistance with DNA testing to establish paternity or law enforcement action to compel fathers to take responsibility Mabetha et al.^3^ The limited involvement of young fathers is further connected to the prevalence of poorly paid, insecure and unprotected work, along with persistent poverty, which hinders men from adequately supporting their families.

Adolescent mothers require significant healthcare support, particularly accessible family planning services. Ntshayintshayi et al.^16^ have expressed a need for these services to be available in their villages, as distance to health facilities and a lack of transport money often prevent access. This aligns with recommendations Ntshayintshayi et al.^16^ to integrate family planning into school settings. Beyond healthcare, social support from partners, parents and the community is also vital. Research from South Africa identifies the male partner as the most important source of support. Male partners, especially those who are also the fathers of the children, are expected to actively support adolescent mothers during pregnancy and postpartum by providing consistent emotional care. The presence of such support fosters a more positive attitude towards pregnancy, offering reassurance and reducing emotional distress.^15^

The findings also indicated that adolescent mothers require substantial educational support to achieve their academic goals. This includes financial aid for school fees and related costs, which is essential for their successful return to education. Adolescent mothers are committed to continuing their studies, as education is key to progress. Support from teachers and access to childcare are also vital to enable their ongoing education.

Some countries have implemented school re-entry policies^17^ to reduce hostile school environments and facilitate their return.^18^ This helps adolescent mothers to go to school and continue with their education. The same policies may be implemented in Qacha’s Nek because they will be beneficial to adolescent mothers.

Adolescent mothers require immediate practical support to meet their basic needs. A lack of access to essential items, such as food and nappies, causes significant distress.^19^ Financial assistance is critical for obtaining these necessities, and support from the fathers of the children is a key resource because both parents are responsible for meeting the needs of their children.^15^ Improving livelihoods through agricultural and informal sector policies is recommended to address the underlying causes of poverty Morgan et al.^20^

Strengthening agricultural policies, such as access to land for farming to ensure household food availability for adolescent mothers, may be helpful to them. Supporting the informal sector, such as small businesses and street vending, can create job opportunities for adolescent mothers and help them to achieve financial autonomy.

The findings highlight the significant benefits of psychosocial support for adolescent mothers, enhancing their overall well-being. This support is essential for meeting daily needs, including nutrition, health and hygiene.

Counselling and psychological interventions also promote peace of mind, reduce stress and improve mental health. Participants reported that overcoming challenges, particularly through employment and financial independence, fosters self-reliance. Ultimately, comprehensive psychosocial support paves the way for a better future for both mother and child, enabling mothers to achieve personal goals and lead more fulfilling lives.

### Strengths and limitations of the study

This study contributes to the body of knowledge by providing an in-depth exploration of the support adolescent mothers need to mitigate the psychosocial challenges they experience after delivery. A limitation is that the study was conducted in only three clinics in the Qacha’s Nek district; thus, the findings cannot be generalised to other health facilities and areas in Qacha’s Nek or Lesotho.

### Recommendations

It is recommended that policies be implemented to integrate the support needs of adolescent mothers into postpartum care. Healthcare providers, including nurses, should be trained in adolescent-sensitive care and counselling. Integrated mental health services should be a core component of postnatal visits. Furthermore, promoting male involvement and co-parenting support is essential to provide practical and psychological assistance to adolescent mothers. Additional research, particularly larger studies that can be generalised, is necessary to evaluate the effectiveness of the various support measures identified by these women in addressing their postpartum psychosocial challenges.

## Conclusion

The study explored adolescent mothers’ perspectives on the support necessary to meet their psychosocial challenges. Adolescent mothers identified a range of support needs, encompassing psychological, social and practical assistance.
